# Supplementary material for: Conditional chemoconnectomics (cCCTomics) as a strategy for efficient and conditional targeting of chemical transmission
Source: eLife. 2024 Apr 30;12:RP91927. doi: 10.7554/eLife.91927 (PMC11060718; doi:10.7554/eLife.91927)
Supplement: Figure 3—source data 3. [file elife-91927-fig3-data3.zip › Figure 3 source data3 part 1.zip!/Figure 3-Source data 3-part1/1082-9-CRISPResso_Report_zod2ewbs.zip!/CRISPResso_on_1082_9/1c.Alignment_barplot.pdf]

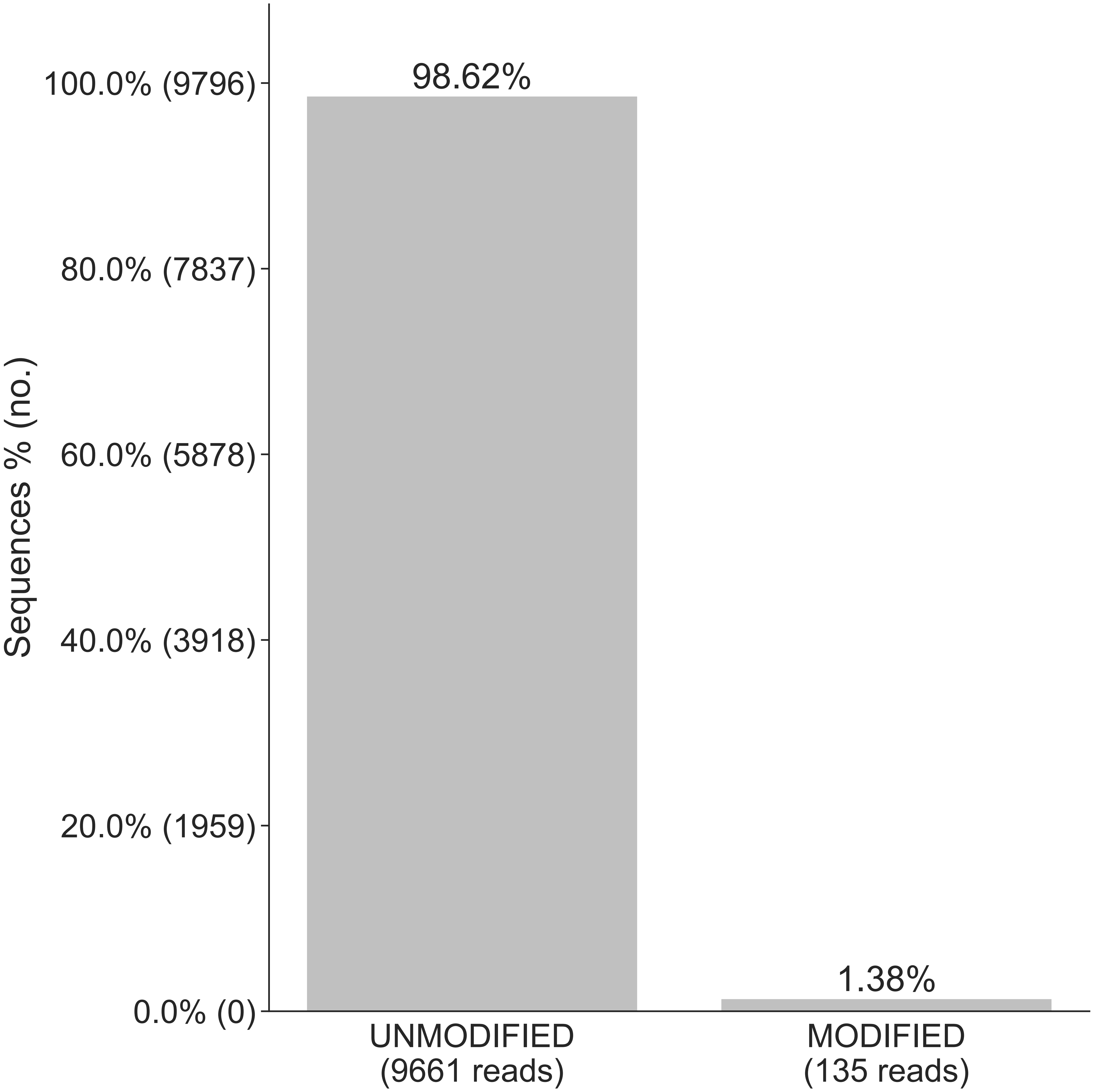
Sequences % (no.)
100.0% (9796)
80.0% (7837)
60.0% (5878)
40.0% (3918)
20.0% (1959)
0.0% (0)
98.62%
1.38%
UNMODIFIED
(9661 reads)
MODIFIED
(135 reads)
